# The Effect of GABAergic Cells Transplantation on Allodynia and Hyperalgesia in Neuropathic Animals: A Systematic Review With Meta-Analysis

**DOI:** 10.3389/fneur.2022.900436

**Published:** 2022-07-04

**Authors:** Zhen-Rong Zhang, Yao Wu, Wen-Jing Wang, Fang-Yong Wang

**Affiliations:** ^1^School of Rehabilitation, Capital Medical University, Beijing, China; ^2^Department of Spine Surgery, China Rehabilitation Research Center, Beijing Bo'ai Hospital, Beijing, China; ^3^Department of Occupational Therapy, China Rehabilitation Research Center, Beijing Bo'ai Hospital, Beijing, China

**Keywords:** GABAergic neurons, cell transplantation, neuropathic pain, mechanical allodynia, heat hyperalgesia

## Abstract

The role of GABAergic cell transplantation in improving neuropathic pain is controversial. We comprehensively searched the relevant literature to identify animal studies of GABAergic cell transplantation that recorded pain behaviors as an outcome according to the Cochrane Handbook 5.0.2. Controlled studies assessing the administration of GABAergic neurons or GABAergic neuronal progenitor cells to rat or mouse neuropathic pain animal models were included. Basic design information and mechanical allodynia thresholds and heat hyperalgesia thresholds data were collected. The risk of bias for the animal experiments was assessed according to the SYRCLE's tool. This study included 10 full-text articles. GABAergic cells transplantation leads to a statistically significant improvement of allodynia (SMD = 5.26; 95% confidence interval: 3.02–7.51; *P* < 0.001) and hyperalgesia (SMD: 4.10; 95% confidence interval: 1.84–6.35; *P* < 0.001). Differentiated GABAergic cells and without antibiotics using may have a better effect for improving neuropathic pain. GABAergic cell transplantation is a promising treatment for improving neuropathic pain. This systematic review and meta-analysis evaluated the effects of GABAergic cell transplantation on neuropathic pain, which can guide future clinical trials and possible clinical treatments, and better attenuate neuropathic pain caused by abnormal circuit hyperexcitability.

## Introduction

Chronic pain can be divided into two categories, inflammatory pain and neuropathic pain. The second is the pain caused by a lesion or disease of the somatosensory nervous system. The prevalence of neuropathic pain is 6.9–10% around the world (1). Drugs such as gabapentin, pregabalin, serotonin/noradrenaline reuptake inhibitors, and tricyclic antidepressants are usually recommended (2), while the long-term effect and tolerance may limit its clinical application. A report on chronic pain in Europe showed that 64% of those taking prescription medicine reported that their pain medication was sometimes inadequate, and 14% had stopped due to side effects (3). In addition to pharmacological treatments, a variety of other interventions are available for neuropathic pain, including intrathecal baclofen bolus, physical and psychological therapies, spinal cord stimulation, surgery, and transcranial magnetic stimulation (4–9). However, due to the complex etiologies of neuropathic pain, current treatments are often inadequate and/or produce severe side effects (10). Scientists must therefore find more efficient and safer solutions to relieve neuropathic pain.

Neuropathic pain is closely related to central sensitization of the spinal dorsal horn, a reduction in the thresholds of cutaneous nociceptors or an increase in the excitability of the central nervous system (CNS) caused by dysfunction of gamma-aminobutyric acid-ergic (GABAergic) neurons in the CNS (11, 12). The central sensitization is closely related to the plasticity of synaptic transmission, and the mechanism may be neuronal sensitization mediated by Glutamate/NMDA receptors (13), glia crosstalk induced by activation of microglia and astrocytes (14, 15), and changes in the (pro-inflammatory) cytokine microenvironment (16). Spinal cord injury (SCI) or peripheral nerve injury (PNI) may negatively affect the function of GABAergic neurons in dorsal horn of the spinal cord, resulting in mechanical allodynia and heat hyperalgesia (17, 18). The recovery of GABAergic neuron function therefore plays an important role in the treatment of pain. It was reported that stem cell transplantation could supplement the inhibitory interneurons in the CNS, restore the function of GABA neurons, and inhibit the mechanical hypersensitivity reaction (19). GABAergic neurons or neural progenitor cells (NPCs) may have similar potential to improve neuropathic pain in SCI or PNI models (20–24). After transplantation into the spinal cord, GABAergic cells can migrate and mainly differentiate into GABAergic intermediate neurons, integrate with the spinal circuit in the deep dorsal horn of the spinal cord (laminae III-V), and may normalize the mechanical threshold and alleviate neuropathic pain (22).

Although some studies analyzed the therapeutic effects of these cells on neuropathic pain in animal models, there was no consistent conclusion or no quantitative data on animal experiments and clinical trials of GABAergic cell therapy for neuropathic pain. Therefore, we comprehensively searched the literature and systematically analyzed animal experimental studies on GABAergic cell transplantation for neuropathic pain, and evaluated the effects of cell transplantation in order to assess the efficacy of GABAergic cell transplantation.

## Methods

### Aims and PICO Statement

This meta-analysis was under the guide of PRISMA Checklist (eMethod 1) and comprehensively retrieved the relevant literature to identify animal experiments of GABAergic cell transplantations and evaluate the effect of these cells on neuropathic pain. The PICO statements are as follows:

1) Population: Neuropathic pain animal models involved rats or mice suffered from SCI or PNI.
2) Intervention: GABAergic cell transplantation.
3) Comparisons: placebo (saline, culture medium, or inactive cells) or no treatment.
4) Outcomes: mechanical allodynia thresholds and heat hyperalgesia thresholds.

### Literature Search Methods

A comprehensive search was conducted to identify all animal experimental studies of GABAergic cell transplantation into models that recorded pain threshold as an outcome according to the Cochrane Handbook 5.0.2 (25). We searched PubMed, Cochrane Library, Web of Science, China Academic Journals Full-text Database, and Wanfang databases for original studies and reviews published with these keywords (“neuropathic pain” OR “neuralgia” OR “allodynia” OR “hyperalgesias” OR “hypersensitivity”) AND (“GABAergic neurons” OR “GABAergic cell” or “neural progenitor cell”) up to March 1, 2021 without language restriction. All titles were independently examined by two reviewers, and any report potentially related was initially included. In addition, we conducted an individual search in the reference lists of relevant articles to find additional studies. We also attempted to contact the authors of the studies with insufficient data to request additional relevant unpublished data. See eMethod 2 for search strategy.

### Inclusion and Exclusion Criteria

This meta-analysis included studies of transplantation of GABAergic neurons or NPCs into SCI or PNI induced neuropathic pain models. Measured outcomes were the evaluation of allodynia and hyperalgesia. Original research studies regarding the influence of transplantation, regardless of donor species or tissue origin, were included.

Any coculture concomitant injection with other cell types, or use of adjuvant products (e.g., matrices, scaffolding), Chemotherapy models, and diabetic neuropathy lead to exclusion. In addition, review articles, commentaries, editorials, letters and experiments without detailed process were excluded.

Two reviewers independently appraised all potentially included studies. Any disagreement was resolved after the third reviewer read through the experiment completely.

### Data Extraction

The data were extracted independently by two reviewers and rechecked after extraction. Any disagreement during the extraction was discussed and resolved. The content included animal characteristics (species, strain, sex, weight or week age), interventions (allogeneic or xenogeneic, delivery route, total number of transplanted cells, degree of cell differentiation, randomization, antibiotic and immunosuppressive usage), effect after the transplantation, and observation (follow-up) time. The data of peak allodynic/hyperalgesic effects after transplantation compared with the control group was extracted as the outcome measures in order to avoid spontaneous recovery over time. The authors were contacted if mean values and standard deviations were not reported. If the information was reported as graphs, we used the method recommended by Sistrom and Mergo to convert raw value of scanned images to original data values from the full text (26).

### Risk of Bias Assessment

The risk of bias (RoB) for the included experiments was assessed according to the Systematic Review Center for Laboratory Animal Experimentation's tool (SYRCLE's tool) (27). Each study was assessed by two reviewers independently and each domain was judged as “low” RoB, “unclear” RoB, or “high” RoB, respectively. Any disagreement was discussed and resolved under the guidance of the third reviewers. According to the recommendation of the SYRCLE's tool, the summary score for each individual study was not calculated for its difficulty to justify the weights assigned (27). Therefore, the sensitivity analysis was not performed.

### Details of Subgrouping

The subgroup analyses were based on the following items:

1) Animal species: Rat models or mice models.
2) Sex: Male animals or female animals.
3) Type of neuropathy: CNS injury (hemisection SCI; contusion SCI, excitotoxic SCI, and compression SCI) or PNI [chronic constriction injury (CCI), spared nerve injury (SNI), spinal nerve ligation (SNL)].
4) Randomization: Yes or no.
5) Transplantation time: Within 2 or 2 weeks after the injury.
6) Delivery route: Intraspinal transplantation or intrathecal transplantation.
7) Graft type: Allogeneic or xenogeneic.
8) Use of antibiotic: Yes or no.
9) Use of immunosuppressive: Yes or no.
10) Number of transplanted cells: <1.5 × 10^6^ cell dose/kg or ≥1.5 × 10^6^ cell dose/kg.
11) Degree of cell differentiation: GABAergic neurons or GABAergic NPCs.

### Statistical Analysis

We used the Review Manager Software package (version 5.3.0; https://training.cochrane.org/online-learning/core-software-cochrane-reviews/revman) and STATA 16.0 (https://www.stata.com/) to conduct the meta-analysis. Effect sizes were computed and the standardized mean difference (SMD) with a 95% confidence interval (CI) was entered in all analyses. By calculating the effect size, and pooling the findings, modifying the bias caused by small sample size was possible.

Statistical heterogeneity among studies and subgroups was evaluated with *I*^2^ test and chi-square tests. If *P* > 0.1 and *I*^2^ < 25%, there was no significant heterogeneity between studies, and a fixed-effect model was used. If P was < 0.1 and *I*^2^ was > 25%, there was likely substantial heterogeneity, and a random effect model was used. The subgroup analyses were adopted to analyze the source of heterogeneity. Due to few included studies, Publication bias has not been evaluated in the subgroup analysis. A two-sided *P*-value < 0.05 was considered statistically significant.

## Results

**Figure 1:**
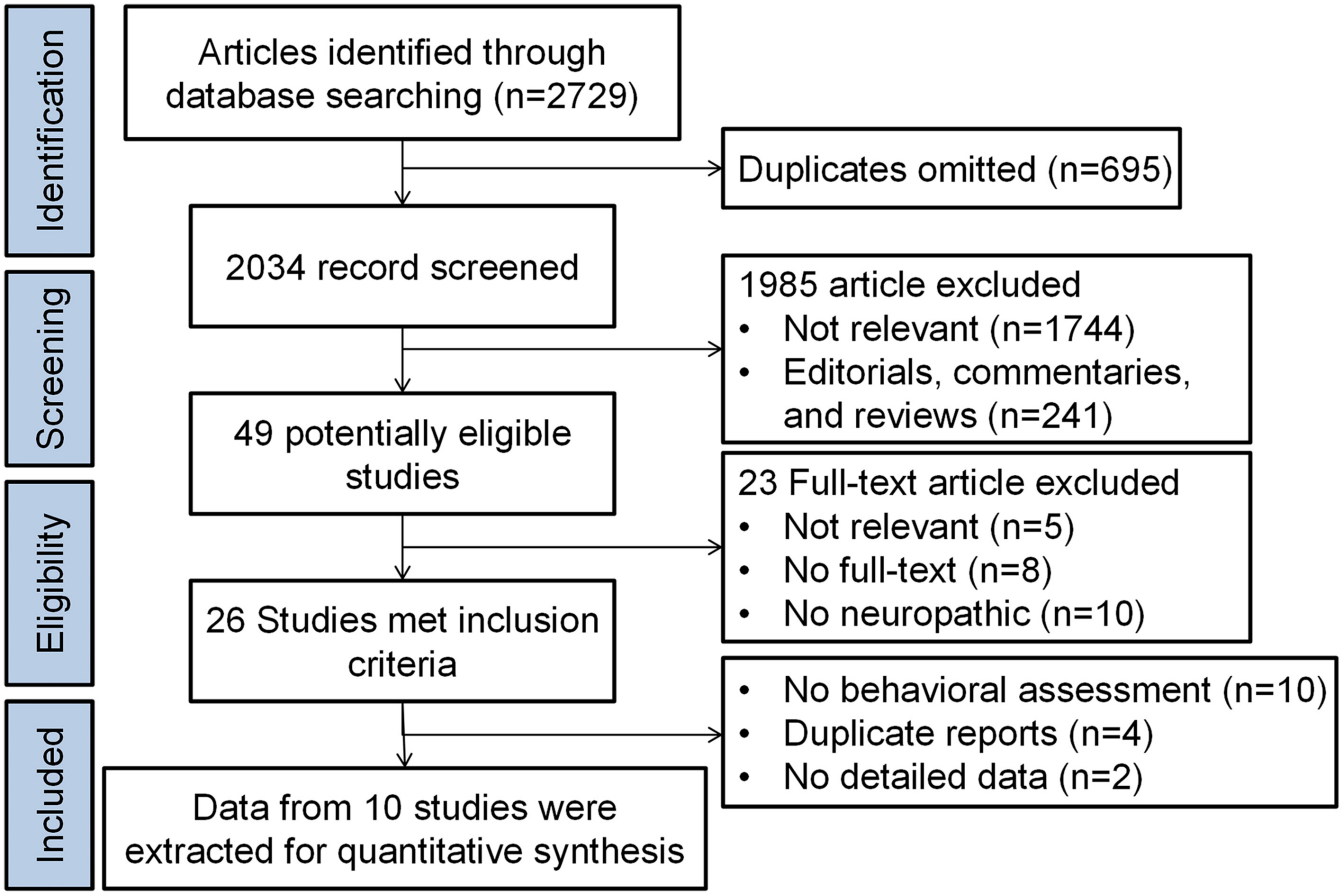
Flow chart of the study.

We found 2,034 unduplicated articles using the search strategies described earlier. Of these, 49 potentially eligible studies were selected for in-depth reading. Excluding irrelevant, republished research (1, 28) and reports that ultimately failed to obtain exact data from papers or authors (22, 24, 29), 10 full-text articles were included for the meta-analysis and were studied in detail as shown in Table 1 (20, 21, 23, 30–36). One studies had raw data (35). The data of other 9 studies were extracted from the pictures by the method recommended by Sistrom and Mergo's (26) and the results of the included reports in the different time intervals was shown in eResult 1. The flow of information from identification to inclusion of studies is summarized in Figure 1. These studies contained a total of 295 rats/mice including 140 GABAergic cell-treated animals and 155 controls. Six studies reported only the impact of GABAergic cell transplantation on mechanical allodynia and four assessed its effects both on mechanical allodynia and heat hyperalgesia.

**Table 1:** Description of the included reports.

**References**	**Sample size**	**Species/Weight or week** **old**	**Model/Intervention**	**Dose/Graft** **type**	**Immunosuppressive/Antibiotic/** **Blinding/Randomization**	**Observation** **time after** **transplantation**
Dugan et al. (30)	12 mMGE-GABAergic NPC/12 vechicle	Male SD rats/250–300 g	Compression SCI /Intraspinal transplantation 4 weeks after injury	2*10^5^ cells/Allogeneic	Yes/No/Yes/Yes	56 days
Eaton et al. (20)	5 hNT2.17 cell-GABAergic neuron/5 vechicle	Male Wistar-Furth rats/200–250 g	Excitotoxic SCI/Intrathecal transplantation 2 weeks after injury	1*10^6^ cells/Xenogeneic	Yes/No/Yes/No	49 days
Fandel et al. (31)	17 hESC-GABAergic neuron/11 vechicle	Female immune-deficient B6.CB17-Prkdc/SzJ mice/12-14 weeks	Contusion SCI/Intraspinal transplantation 2 week after SCI	4.5*10^5^ cells/Xenogeneic	No/Yes/Yes/Yes	180 days
Hwang et al. (32)	7 mESC-GABAergic NPC/7 vechicle	Male Sprague–Dawley rats/200–220 g	Contusion SCI/Intrathecal transplantation 3 weeks after injury	1*10^6^ cells/Xenogeneic	Yes/Yes/Yes/Yes	49 days
Jergova et al. (23)	6 GABAergic NPC/6 vechicle	Male SD rats/140–160 g	CCI of sciatic nerve/Intraspinal transplantation 1 weeks after injury	3*10^5^ cells/Allogeneic	Yes/No/Yes/No	28 days
Jergova et al. (33)	10 GABAergic NPC/6 vechicle	Male SD rats/140–160 g	Compression SCI/Intraspinal transplantation 5 weeks after injury	2*10^5^ cells/Allogeneic	Yes/Yes/Yes/No	42 days
Kim et al. (34)	40 mESC-GABAergic neuron/76 vehicle	Male SD rats/170–200 g	Hemisection SCI/Intrathecal transplantation 2 weeks after SCI	5*10^5^ cells/Xenogeneic	Yes/No/Yes/No	70 days
Li (35)	5 hESC-GABAergic NPC/5 vechicle	Male SD rats/200–220 g	Contusion SCI/Intraspinal transplantation 1 weeks after injury	1*10^6^ cells/Xenogeneic	Yes/Yes/Yes/Yes	28 days
Manion et al. (36)	29 human iPS-GABAergic neuron/21 vechicle	Male NOD prkdSCID mice/10 weeks	SNI/Intraspinal transplantation 1 weeks after injury	2*10^5^ cells/Xenogeneic	No/Yes/Yes/Yes	56 days
Mukhida et al. (21)	7 human GABAergic/5 vechicle	Female Wistar rats/175–200 g	SNL/Intraspinal transplantation 10 days post-ligations	2*10^5^ cells/Xenogeneic	Yes/No/Yes/Yes	42 days

*CCI, chronic constriction injury; hESC, human embryonic stem cell; mESC, mouse embryonic stem cell; mMGE, mouse medial ganglion eminence; NPC, neural progenitor cell; SCI, spinal cord injury; SD rats, Sprague–Dawley rats; SNI, spared nerve injury; SNL, spinal nerve ligation*.

### Heterogeneity

According to the results of the therapeutic effects of GABAergic cells, a significant statistical heterogeneity was found with allodynia (*I*^2^: 95%; P < 0.001) and hyperalgesia (*I*^2^: 85%; *P* < 0.001). Therefore, in these cases a random-effect model was used. In addition, we were not able to calculate the pooled-effect size in mice, female animals or experiments without immunosuppression because there were only two eligible studies.

### Meta-Analysis

**Figure 2:**
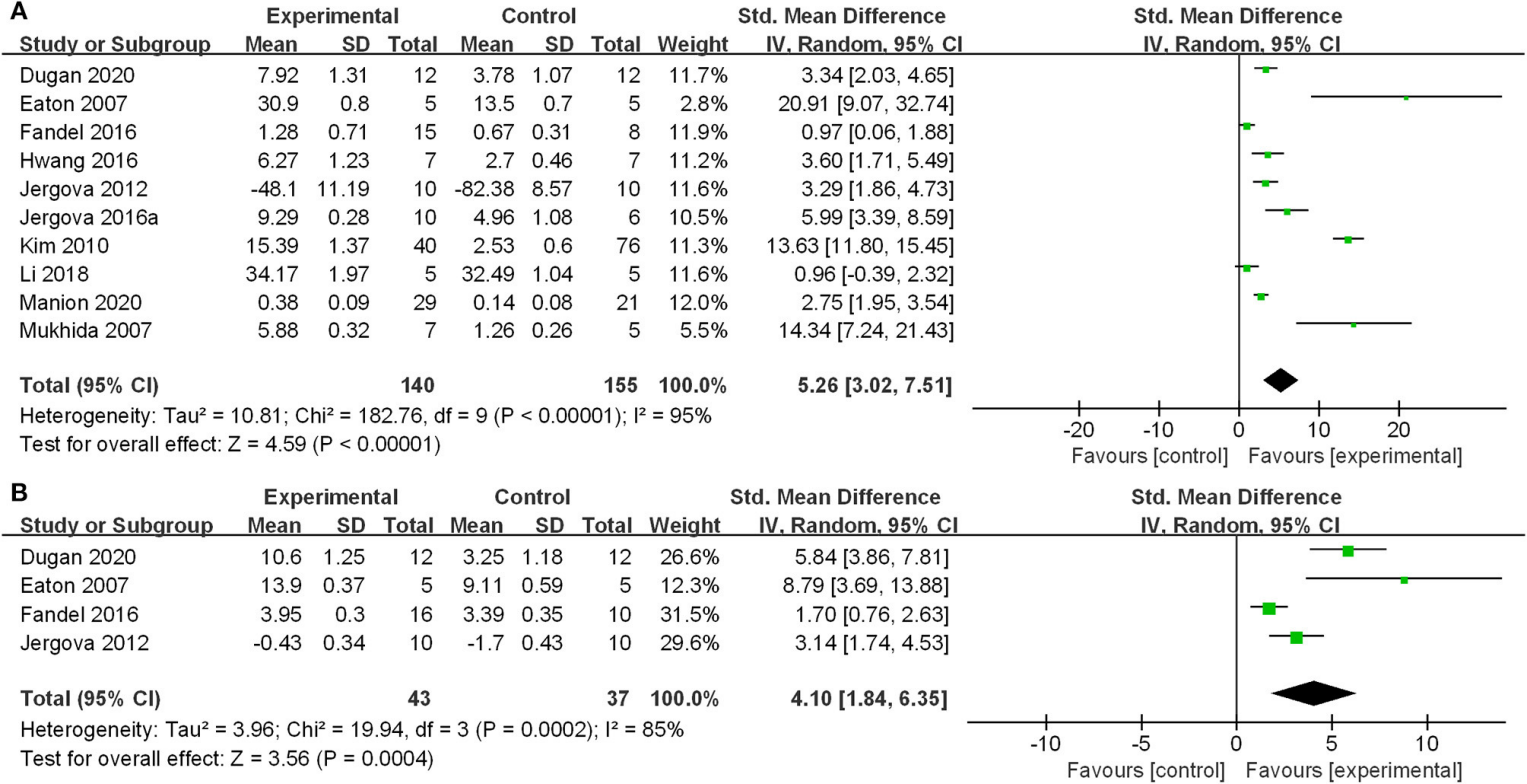
Effect of GABAergic cells on mechanical allodynia and heat hyperalgesia. **(A)** Mechanical allodynia; **(B)** Heat hyperalgesia. GABAergic cell transplantation attenuated the mechanical allodynia and heat hyperalgesia of the animal models. The standardized mean differences were 5.26 (95% CI: 3.02–7.51) and 4.10 (95% CI: 1.84–6.35), respectively.

The main outcome measure was the assessment of mechanical allodynia and heat hyperalgesia thresholds. According to our analyses, using the random-effects model, GABAergic cell transplantation leads to a statistically significant improvement of mechanical allodynia (SMD: 5.26; 95% CI: 3.02–7.51) and a significant effect on heat hyperalgesia (SMD: 4.10; 95% CI: 1.84–6.35). Forest plots of the effects of GABAergic cells on mechanical allodynia and heat hyperalgesia are shown in Figure 2.

### Subgroup Analyses of Mechanical Allodynia

Subgroup analyses of mechanical allodynia thresholds were performed based on the above items. Due to the small number of studies included (four studies), subgroup analyses of heat hyperalgesia were not done.

Table 2 shows the subgroup analysis of allodynia thresholds. Multivariate meta-regression analysis showed that randomization (*P* = 0.04), antibiotic (*P* = 0.01), and the cell type (*P* = 0.03) influenced the improvement of mechanical allodynia after GABAergic cell transplantation. As shown in the Table 2, no differences were found in the effect of GABAergic cells transplantation on allodynia in PNI model or CNS injury model (*P* = 0.46), delivery route (*P* = 0.06), varying the transplantation time (*P* = 0.17) and the number of transplanted cells (*P* = 0.54).

**Table 2:** Subgroup analyses of the effect of GABAergic cells on mechanical allodynia.

**Characteristic**	**Model**	***P* (I^2^)^a^**	**Effect size (95%CI)^b^**	***P***	**Subgroup differences**
Animal spices					NA
Mice	NA	NA	NA	NA	
Rat	REM	<0.001 (95%)	6.72 (3.44, 10.00)	<0.001	
Sex					NA
Male	REM	<0.001 (95%)	5.32 (2.73, 7.92)	<0.001	
Female	NA	NA	NA	NA	
Type of neuropathy					0.46
CNS injury	REM	<0.001 (97%)	5.60 (2.18, 9.02)	0.001	
PNI	REM	0.006 (81%)	4.03 (1.68, 6.37)	<0.001	
Randomization					0.04
No	REM	<0.001 (96%)	9.57 (3.20, 15.94)	0.003	
Yes	REM	<0.001 (82%)	2.64 (1.31, 3.97)	<0.001	
Transplantation time					0.17
Within 2 weeks	REM	<0.001 (86%)	2.94 (1.23, 4.64)	<0.001	
After 2 weeks	REM	<0.001 (97%)	6.63 (1.67, 11.60)	<0.001	
Delivery route					0.06
Intraspinal	REM	<0.001(83%)	3.02 (1.71, 4.33)	<0.001	
Intrathecal	REM	<0.001(97%)	11.41 (2.71, 20.12)	0.01	
Graft type					0.19
Xenogeneic	REM	<0.001 (97%)	6.17 (2.90, 9.44)	<0.001	
Allogeneic	REM	0.17 (44%)	3.81 (2.53, 5.10)	<0.001	
Use of antibiotic					0.01
No	REM	<0.001 (96%)	9.57 (4.31, 14.83)	<0.001	
Yes	REM	<0.001 (82%)	2.54 (1.18, 3.90)	<0.001	
Use of immunosuppressive					NA
No	NA	NA	NA	NA	
Yes	REM	<0.001 (95%)	6.72 (3.44, 10.00)	<0.001	
Number of transplanted cells					0.54
<1.5 x 10^6^ cell dose/kg	REM	<0.001 (85%)	6.39 (2.42, 10.36)	<0.001	
More than or equal to 1.5 x 10^6^ cell dose/kg	REM	<0.001 (96%)	4.86 (2.02, 7.69)	0.003	
Degree of cell differentiation					0.03
GABAergic neuron	REM	<0.001 (98%)	11.95 (3.76,20.13)	0.004	
GABAergic NPC	REM	<0.001 (81%)	2.80 (1.46, 4.14)	<0.001	

*NA, not applicable; NPC, neural progenitor cell; REM, random effect model*.

a *Heterogeneity among studies*.

b *Standardized mean difference*.

### Risk of Bias Assessment

All domains of these studies were marked as low or unclear RoB according to the SYRCLE's tool. Because some circumstances or randomization sequences were not clearly described, and could not be judged based on the information, 56.0% of the entries were rated as “unclear RoB.” See Table 3 (and eResult 2 for support information for judgement) for details.

**Table 3:** The risk of bias in the included studies.

**Item**	**Type of bias**	**Domain**	**Dugan** **et al. (30)**	**Eaton** **et al. (20)**	**Fandel** **et al. (31)**	**Hwang** **et al. (32)**	**Jergova** **et al. (23)**	**Jergova** **et al. (33)**	**Kim et al.** **(34)**	**Li** **(35)**	**Manion** **et al. (36)**	**Mukhida** **et al. (21)**
1	Selection bias	Sequence generation	Unclear	Unclear	Low	Low	Unclear	Unclear	Unclear	Unclear	Unclear	Unclear
2		Baseline characteristics	Unclear	Unclear	Low	Unclear	Unclear	Unclear	Unclear	Unclear	Unclear	Unclear
3		Allocation concealment	Unclear	Unclear	Low	Unclear	Unclear	Unclear	Unclear	Unclear	Low	Unclear
4	Performance bias	Random housing	Unclear	Low	Low	Low	Unclear	Low	Low	Low	Low	Low
5		Blinding	Unclear	Unclear	Low	Unclear	Unclear	Unclear	Unclear	Unclear	Low	Unclear
6	Detection bias	Random outcome assessment	Unclear	Unclear	Unclear	Unclear	Unclear	Unclear	Unclear	Unclear	Unclear	Unclear
7		Blinding	Low	Low	Low	Low	Low	Low	Low	Low	Low	Low
8	Attrition bias	Incomplete outcome data	Unclear	Unclear	Low	Low	Unclear	Unclear	Unclear	Unclear	Low	Low
9	Reporting bias	Selective outcome reporting	Low	Unclear	Low	Low	Low	Low	Low	Unclear	Low	Low
10	Other bias	Other sources of bias	Unclear	Unclear	Low	Low	Low	Low	Low	Unclear	Low	Low

*Support for judgement were shown in the eResult 2*.

## Discussion

The cell repair research in recent years mainly focuses on the restoration of motor function (37–40), but rarely involves in improving neuropathic pain. Most preclinical studies were conducted on rodents and involve direct nerve injury, often to the sciatic nerve or spinal cord. These models bear poor resemblance to the case history of most neuropathic pain patients, but they do enable disease processes to be investigated, and some treatments are able to reverse the pain behaviors (41). Although these models had their limitations, and translation was currently far from perfect, such models have identified changes at all levels in the pain pathway in neuropathic pain, including alterations in sensory neuron protein expression, changes in spinal cord synaptic function and descending control of pain from the brain (42).

We found that although both GABAergic neurons and GABAergic NPCs can improve neuropathic pain, direct transplantation with GABAergic neurons was more effective than cell therapy with GABAergic NPCs. This may be due to the fact that NPCs can only function if they differentiate into GABAergic cells, but will not be effective when they differentiate into glial cells (21). And transplanted cell had different fates in differentiation, which was related to the type of cells transplanted. In Braz's and Hwang's studies, transplanted GABAergic neuron precursors mainly differentiated into neurons rather than microglia and astrocytes (22, 32). In Fandel's and Kim's study, transplanted hESC-MGEs migrate within the injured spinal cord and mainly differentiating into GABAergic neuron subtypes and glial cells (31, 34). However, different transplanted cells mainly integrated with the spinal circuit in the deep dorsal horn of the spinal cord (laminae III-V) (22, 23). Transplanted GABAergic cells can survive in the spinal cord, and alleviate mechanical allodynia and heat hyperalgesia in neuropathic pain models.

According to existing cell transplantation clinical trials to treat SCI, we found that the cell doses were approximately 1.5 × 10^6^ cell dose/kg (43), 1.8 × 10^6^ cell dose/kg (44), and 4.5 × 10^6^ cell dose/kg (45), assuming that all patients weighed 67 kg. So we evaluated the effect of cell dose on the neuropathic pain model using 1.5 x 10^6^ cell dose/kg as the cut-off value. However, after dividing by this value, both high and low doses have effectively alleviated neuropathic pain and no statistical difference were found.

Our results indicated that with or without antibiotics, both treatments could relieve neuropathic pain, and animals that did not use antibiotics received better mechanical allodynia relief. The five articles reported the use of antibiotics, and all were injected after injury. Because the number was too small, effective analysis was difficult. Another meta-analysis showed that olfactory ensheathing cell transplantation had the same effect with or without antibiotics (46). This result could stimulate the interest of other scientists and promote further studies regarding the use of antibiotics in animals after transplantation. Xenogeneic cell transplantation may improve neuropathic pain more effectively than allogeneic cell transplantation. This was caused by the difference of animal species in this meta-analysis for animal models were mainly rats while the transplanted cells were mainly from humans and mice.

Some studies did not report or analyze the measured outcomes at the same time points, which led to the design of this meta-analysis only analyzed the peak effects of cell transplantation, rather than the results at the same time point after transplantation. The meta-analysis of some cells such as bone marrow-derived mesenchymal stem cells and olfactory ensheathing cells had proved the long-term effect of alleviating neuropathic pain after cell transplantation (46, 47). The time of nociceptive testing post-transplantation varied greatly, such as time intervals (for example, weekly, bi-weekly), and the longest follow-up time is also inconsistent (see Table 1; eResult 1). The responsiveness to mechanical and thermal stimuli may alter over time, perhaps due to the changes in cell viability, spontaneous recovery following injury, or dynamic reorganization, which would also limit the promotion of this meta-analysis (31, 48).

The SYRCLE's tool, based on the Cochrane Collaboration RoB Tool, aims to assess methodological quality and has been adapted to aspects of bias and plays a role in animal experiments (27). However, several previous meta-analyses of cell transplantation to alleviate neuropathic pain have not analyzed RoB based on the SYRCLE's tool (19, 46, 47). Another systematic review evaluated the therapeutic potential of regulatory T lymphocytes in periodontitis animal models and rated 42.11% of projects as unclear RoB (49). In future studies, it is strongly recommended that animal experiments of alleviating neuropathic pain should refer to some checklist to improve the quality control, for example, the guidelines of Planning Research and Experimental Procedures on Animals: Recommendations for Excellence (48).

Drew et al. found that mechanical allodynia in rats after SCI was related to the loss of GABAergic inhibition in the dorsal horn (17). Kim et al. (34) confirmed the major cause of pain was the loss of the spinal GABAergic system following SCI. This provided a theoretical basis for treatment of neuralgia using GABAergic neuron transplantation. Thus, transplantation of GABAergic neurons or GABAergic NPCs may have effects on mechanical allodynia and heat hyperalgesia. Llewellyn-Smith et al. (48) transplanted GFP-expressing MGE derived neuronal precursors into mice with SCI and found that cells directly integrated into the intact dorsal horn circuitry and the transplanted MGE neurons retained their GABAergic phenotypes and integrated dynamically into host-transplant synaptic circuits. These transplanted cells developed into mature neurons, and exhibited a specific discharge pattern of cortical and spinal cord inhibitory interneurons, and integrated with the host circuit (24, 32). Integrated cells alleviated neuropathic pain through synaptic release of GABA in the spinal cord and affected the intrathecal spinal environment for sensory system modulation (25, 50). These results illustrate the remarkable plasticity of spinal cord and the potential of cell-based therapy in human.

After PNI, the proportion of GABA-immunoreactive neurons in the spinal dorsal horn on the nerve-injured side was reduced (51), which may disrupt GABAergic inhibition in spinal dorsal horn and cause some forms of neuropathic pain (23, 52). These included studies did not show a difference in effects comparing with SCI models, indicating that GABAergic cells are equally effective in neuropathic pain caused by PNI. Manion et al. observed that the transplanted GABAergic neurons also survived for a long time in the uninjured spinal cord and integrate with the original synapses (36). These transplanted cells may restore GABAergic function of the spinal cord dorsal horn to reduce the hyperexcitability and exaggerate dorsal horn neuronal firings that develop in dorsal horn projection neurons (21, 23).

Although, similar study had been published (53), our meta-analysis is not redundant. We set stricter inclusion and exclusion criteria to ensure the consistency of studies, the included studies were not completely consistent, and sensitivity and publication bias could not be assessed. It should be noted that the meta-analysis conducted by Askarian-Amiri S et al. appears to be loose on the risk of bias assessment, and two studies were not included (30, 35). The evaluation basis can be inquired in the attachment. Finally, there is no doubt about the benefit of GABAergic neuron transplantation in improving neuropathic pain in animals. Therefore, we focused on the mechanism of cell transplantations in improving neuropathic pain and the limitations of these studies, and also conducted the subgroup analysis for the included experiments.

In the literature we searched, there was no such clinical trial on GABAergic cell transplantation to treat human neuropathic pain, even after we specifically searched the literature on human trials. Therefore, it is difficult to summarize the potential risks of intraspinal GABAergic cell transplantation. These animals involved were rats or mice, and no experiments were found in other mammals. The study by Fandel et al. (31) showed that transplantation of MGE-like precursor cells in the normal mouse spinal cord did not alter spinal cord function. Another study showed that transplantation of well-differentiated GABAergic cell into the central nervous system did not form tumors in rats (20). Among the included studies, 56.0% were rated as unclear RoB, which posed a challenge for the further trials of these cells. The publication bias results indicated that more research was needed to supplement the evidence for the effect of GABAergic neurons on neuropathic pain relief. Although there are no clinical trials that specifically applied GABAergic cells to improve neuropathic pain behaviors, this meta-analysis suggests that a combination of multiple cell transplantations may be necessary to achieve maximum benefits.

## Limitations

Although the methodological quality of the 10 included trials had low risk bias, the number of trials examined was small because of different animal species, type of neuropathy, cell types/origins, cell quantities transfused, and transplantation times after SCI; thus, the heterogeneity between each study was high and may lead to a far greater effect size. This meta-analysis therefore had a potential RoB and only four studies involved to thermal reflex hypersensitivity, which prompts the need to avoid the risk for over-speculation. In addition, some of the included studies did not list how many animals were excluded from testing. Consequently, additional studies with larger sample sizes, longer-term outcome measurements, and more detailed observations and explanations are needed to validate the findings described in this meta-analysis. Few included reports, limited reporting of treatment effect estimates and unclear RoB suggest using caution when assessing the effects of GABAergic cells transplantation.

## Conclusion

GABAergic cell transplantation is a very promising treatment for attenuating neuropathic pain. Our meta-analysis showed that GABAergic cells can partially alleviate mechanical allodynia and heat hyperalgesia after SCI or PNI. Intrathecal transplantation, a xenogeneic cell source, differentiated GABAergic cells, and no antibiotics may be better for improving neuropathic pain. It is necessary to reduce the RoB and improve the quality of animal experiments. This will guide future clinical trials and possible clinical treatments, and better alleviate neuropathic pain caused by abnormal circuit hyperexcitability.

## Data Availability Statement

The original contributions presented in the study are included in the article/Supplementary Material, further inquiries can be directed to the corresponding author.

## Author Contributions

Material preparation, data collection and analysis were performed by Z-RZ, F-YW, and YW. The first draft of the manuscript was written by Z-RZ and all authors commented on previous versions of the manuscript. All authors contributed to the study conception and design, read, and approved the final manuscript.

## Funding

This work was funded by National Key R&D Program of China (Project No. 2021YFF0501600 and Subject No. 2021YFF0501604) and Capital's Funds for Health Improvement and Research (2022-2-6013).

## Conflict of Interest

The authors declare that the research was conducted in the absence of any commercial or financial relationships that could be construed as a potential conflict of interest.

## Publisher's Note

All claims expressed in this article are solely those of the authors and do not necessarily represent those of their affiliated organizations, or those of the publisher, the editors and the reviewers. Any product that may be evaluated in this article, or claim that may be made by its manufacturer, is not guaranteed or endorsed by the publisher.

## Supplementary Material

eMethod 1 PRISMA Checklist.

eMethod 2 Search strategy of every database.

eResult 1 Data set of the included reports.

eResult 2 Support for judgement the SYRCLE's risk of bias tool.

## Abbreviations

CCI: chronic constriction injury
CI: confidence interval
CNS: central nervous system
GABA: gamma-aminobutyric acid
hESC: human embryonic stem cell
mESC: mouse embryonic stem cell
mMGE: mouse medial ganglion eminence
NPC: neural progenitor cell
PNI: peripheral nerve injury
RoB: risk of bias
SCI: spinal cord injury
SD rats: Sprague–Dawley rats
SMD: standardized mean difference
SNI: spared nerve injury
SNL: spinal nerve ligation
SYRCLE's tool: Systematic Review Center for Laboratory Animal Experimentation's tool.
