# Supplementary material for: The Effect of GABAergic Cells Transplantation on Allodynia and Hyperalgesia in Neuropathic Animals: A Systematic Review With Meta-Analysis
Source: Front Neurol. 2022 Jul 4;13:900436. doi: 10.3389/fneur.2022.900436 (PMC9289294; doi:10.3389/fneur.2022.900436)
Supplement: eMethod 2 — Search strategy of every database. [file Data_Sheet_2.docx]

**eMethod 2. search strategy of every database**

Search Date: March 1, 2021

Search scope: all sub-databases in the database.

| Database | strategy | Results |
| --- | --- | --- |
| PubMed | MESH: GABAergic Neurons Entry Terms: GABAergic Neuron or Neuron, GABAergic or Neurons, GABAergic or GABA Cells or Cell, GABA or Cells, GABA or GABA Cell or GABA Neurons or GABA Neuron or Neuron, GABA or Neurons, GABA | - |
|  | MeSH: Neuralgia  Entry Terms: Neuralgias or Neuropathic Pain or Neuropathic Pains or Pain, Neuropathic or Pains, Neuropathic or Neurodynia or Neurodynias or Nerve Pain or Nerve Pains or Pain, Nerve or Pains, Nerve or neuropathic pain or allodynia or Hyperalgesias or Hyperalgesic Sensations or hypersensitivity | - |
|  | (neuralgia or neuralgia or neuropathic pain or neuropathic pains or pain, neuropathies or pains, neuropathies or neurodynia or neurodynia or neve pain or nerve pain or pain, Nerve or Pains, nerve or neuropathic pain or allodynia or hyperalgesia or hyperalgesic sensation or hypersensitivity) AND (GABAergic Neurons or GABAergic Neuron or Neuron, GABAergic or Neurons, GABAergic or GABA Cells or Cell, GABA or Cells, GABA or GABA Cell or GABA Neurons or GABA Neuron or Neuron, GABA or Neurons, GABA) | 901 |
| Cochrane | Title Abstract Keyword: Neuralgias or Neuropathic Pain or Neuropathic Pains or Pain, Neuropathic or Pains, Neuropathic or Neurodynia or Neurodynias or Nerve Pain or Nerve Pains or Pain, Nerve or Pains, Nerve or neuropathic pain or allodynia or Hyperalgesias or Hyperalgesic Sensations or hypersensitivity  AND Title Abstract Keyword: GABAergic Neuron or Neuron, GABAergic or Neurons, GABAergic or GABA Cells or Cell, GABA or Cells, GABA or GABA Cell or GABA Neurons or GABA Neuron or Neuron, GABA or Neurons, GABA | 10 |
| Web of Science Database = WOS, BIOSIS, KJD, MEDLINE, RSCI, SCIELO | # 1=78,017  TS=(GABAergic Neuron or Neuron, GABAergic or Neurons, GABAergic or GABA Cells or Cell, GABA or Cells, GABA or GABA Cell or GABA Neurons or GABA Neuron or Neuron, GABA or Neurons, GABA) | - |
|  | # 2=369,681  TS=(Neuralgias or Neuropathic Pain or Neuropathic Pains or Pain, Neuropathic or Pains, Neuropathic or Neurodynia or Neurodynias or Nerve Pain or Nerve Pains or Pain, Nerve or Pains, Nerve or neuropathic pain or allodynia or Hyperalgesias or Hyperalgesic Sensations or hypersensitivity) | - |
|  | # 3= #2 AND #1 | 1688 |
| China Academic Journals Full-text Database | 主题：GABA能细胞 + GABA能神经元  并且主题：神经病理性疼痛 + 痛觉过敏  选择“中英文扩展”  Theme: GABAergic cells or GABAergic neurons  And theme: neuropathic pain or hyperalgesia  Select "Chinese and English Extension" | 79 |
| Wanfang Data Database | 检索表达式（中英文扩展&主题词扩展）： 主题:(GABA能神经元 or GABA能细胞)*主题:(神经病理性疼痛)  Search expression (Chinese and English expansion & subject term expansion): Subject: (GABAergic neuron or GABAergic cell) * Subject: (Neuropathic pain) | 47 |
