# Supplementary material for: The Effect of GABAergic Cells Transplantation on Allodynia and Hyperalgesia in Neuropathic Animals: A Systematic Review With Meta-Analysis
Source: Front Neurol. 2022 Jul 4;13:900436. doi: 10.3389/fneur.2022.900436 (PMC9289294; doi:10.3389/fneur.2022.900436)
Supplement: eResult 1 — Data set of the included reports. [file Data_Sheet_3.docx]

**eResult 1. date set of the included reports**

The bold font in the table indicates that the effect is the largest at this follow-up time point, which is included in the calculation.

**1. Mechanical allodynia threshold (g)**

**Dugan 2020**

| Days after transplantation | Experiment | | | Control | | |
| --- | --- | --- | --- | --- | --- | --- |
|  | Mean | SD | N | Mean | SD | N |
| -28 (pre-injury) | 14.40 | NA | 12 | 14.82 | NA | 12 |
| -21 | 13.95 | NA | 12 | 14.95 | NA | 12 |
| -14 | 12.00 | 1.11 | 12 | 11.13 | 1.26 | 12 |
| -7 | 5.31 | 1.15 | 12 | 6.94 | 1.20 | 12 |
| 0 | 4.57 | 1.20 | 12 | 6.13 | 1.20 | 12 |
| 7 | 6.08 | 0.83 | 12 | 5.23 | 1.31 | 12 |
| 14 | 6.26 | 0.87 | 12 | 4.49 | 1.08 | 12 |
| 21 | 6.31 | 0.78 | 12 | 4.49 | 1.07 | 12 |
| 28 | 7.40 | 1.41 | 12 | 3.83 | 1.20 | 12 |
| 35 | 7.82 | 1.18 | 12 | 3.78 | 1.22 | 12 |
| 42 | 7.87 | 1.04 | 12 | 4.12 | 1.37 | 12 |
| **49** | **7.92** | **1.31** | **12** | **3.78** | **1.07** | **12** |
| 56 | 7.39 | 1.6 | 12 | 3.72 | 1.12 | 12 |

**Eaton 2007**

| Days after transplantation | Experiment | | | Control | | |
| --- | --- | --- | --- | --- | --- | --- |
|  | Mean | SD | N | Mean | SD | N |
| -14 (pre-injury) | 32.14 | NA | 5 | 28.04 | 12.74 | 5 |
| 0 | 15.83 | 1.00 | 5 | 17.05 | 1.02 | 5 |
| 7 | 25.61 | 1.08 | 5 | 16.00 | NA | 5 |
| 14 | 26.83 | 0.91 | 5 | 16.26 | NA | 5 |
| 21 | 26.88 | 1.15 | 5 | 14.86 | 0.44 | 5 |
| 28 | 27.52 | 1.26 | 5 | 14.26 | 0.67 | 5 |
| 35 | 27.26 | 1.18 | 5 | 13.52 | 0.57 | 5 |
| 42 | 27.09 | 0.87 | 5 | 13.09 | 0.48 | 5 |
| **49** | **30.9** | **0.8** | **5** | **13.5** | **0.7** | **5** |

**Fandel 2016**

| Days after transplantation | Experiment | | | Control | | |
| --- | --- | --- | --- | --- | --- | --- |
|  | Mean | SD | N | Mean | SD | N |
| -14 (Pre-injury) |  |  |  | 0.93 | 0.25 | 11 |
| 0 |  |  |  | 0.56 | 0.43 | 10 |
| 90 | 0.78 | 0.09 | 17 | 0.53 | 0.11 | 11 |
| **180** | **1.28** | **0.71** | **15** | **0.67** | **0.31** | **8** |

**Hwang 2016**

| Days after transplantation | Experiment | | | Control | | |
| --- | --- | --- | --- | --- | --- | --- |
|  | Mean | SD | N | Mean | SD | N |
| -21 (pre-injury) | 14.10 | 0.54 | 7 | 13.77 | 0.50 | 7 |
| 7 | 2.53 | 0.26 | 7 | 2.16 | 0.46 | 7 |
| 14 | 2.85 | 0.46 | 7 | 2.81 | 0.35 | 7 |
| 21 | 3.81 | 0.61 | 7 | 3.11 | 0.26 | 7 |
| **28** | **6.27** | **1.23** | **7** | **2.70** | **0.46** | **7** |
| 35 | 5.76 | 0.57 | 7 | 3.17 | 0.35 | 7 |
| 42 | 6.02 | 1.23 | 7 | 3.24 | 0.29 | 7 |
| 49 | 6.09 | 1.26 | 7 | 3.10 | 0.30 | 7 |

**Jergova 2012**

| Days after transplantation | Experiment | | | Control | | |
| --- | --- | --- | --- | --- | --- | --- |
|  | Mean | SD | N | Mean | SD | N |
| -7 (pre-injury) | -10.48 | 9.29 | 10 | -10.48 | 9.29 | 10 |
| 0 | -50.95 | 6.43 | 10 | -50.95 | 6.43 | 10 |
| 7 | -66.19 | 11.19 | 10 | -80.71 | 11.19 | 10 |
| 14 | -38.33 | 4.05 | 10 | -62.14 | 5.48 | 10 |
| **21** | **-48.10** | **11.19** | **10** | **-82.38** | **8.57** | **10** |
| 28 | -50.71 | 8.81 | 10 | -62.14 | 5.71 | 10 |

**Jergova 2016a**

| Days after transplantation | Experiment | | | Control | | |
| --- | --- | --- | --- | --- | --- | --- |
|  | Mean | SD | N | Mean | SD | N |
| -35 (pre-injury) | 13.29 | 1.54 | 10 | 14.89 | 0.22 | 6 |
| -28 | 6.48 | 0.94 | 10 | 6.62 | 1.13 | 6 |
| -21 | 5.32 | 1.02 | 10 | 4.52 | 0.55 | 6 |
| -14 | 5.24 | 1.16 | 10 | 5.29 | 0.69 | 6 |
| -7 | 5.29 | 2.95 | 10 | 6.26 | 1.35 | 6 |
| 0 | 5.05 | 1.10 | 10 | 4.22 | 0.47 | 6 |
| 7 | 6.45 | 0.83 | 10 | 3.89 | 0.47 | 6 |
| 14 | 6.42 | 1.27 | 10 | 3.56 | 0.36 | 6 |
| **21** | **9.29** | **0.28** | **10** | **4.96** | **1.08** | **6** |
| 28 | 7.94 | 0.52 | 10 | 4.55 | 0.85 | 6 |
| 35 | 7.31 | 1.21 | 10 | 5.32 | 0.85 | 6 |
| 42 | 6.87 | 0.28 | 10 | 5.63 | 1.27 | 6 |

**Kim 2010**

| Days after transplantation | Experiment | | | Control | | |
| --- | --- | --- | --- | --- | --- | --- |
|  | Mean | SD | N | Mean | SD | N |
| -14 (pre-injury) | 18.07 | 0.24 | 40 | 18.10 | 0.24 | 76 |
| -7 | 8.90 | 1.81 | 40 | 8.55 | 2.17 | 76 |
| 0 | 2.21 | 0.24 | 40 | 2.08 | 0.29 | 76 |
| 7 | 2.26 | 0.24 | 40 | 1.70 | 0.24 | 76 |
| 14 | 2.53 | 0.38 | 40 | 1.90 | 0.33 | 76 |
| 21 | 4.20 | 1.10 | 40 | 1.97 | 0.37 | 76 |
| 28 | 7.09 | 1.58 | 40 | 1.99 | 0.51 | 76 |
| 35 | 9.32 | 1.72 | 40 | 2.08 | 0.33 | 76 |
| 42 | 14.58 | 1.40 | 40 | 2.06 | 0.24 | 76 |
| **49** | **15.39** | **1.37** | **40** | **2.53** | **0.60** | **76** |
| 56 | 15.01 | 1.46 | 40 | 3.36 | 0.78 | 76 |
| 63 | 15.18 | 1.25 | 40 | 3.04 | 0.63 | 76 |
| 70 | 14.9 | 1.40 | 40 | 2.50 | 0.52 | 76 |

**Li 2018**

| Days after transplantation | Experiment | | | Control | | |
| --- | --- | --- | --- | --- | --- | --- |
|  | Mean | SD | N | Mean | SD | N |
| 28 | 34.17 | 1.97 | 5 | 32.49 | 1.04 | 5 |

**Manion 2020**

| Days after transplantation | Experiment | | | Control | | |
| --- | --- | --- | --- | --- | --- | --- |
|  | Mean | SD | N | Mean | SD | N |
| -7 (pre-injury) | 0.62 | 0.03 | 29 | 0.63 | 0.03 | 21 |
| -1 | 0.09 | 0.01 | 29 | 0.07 | 0.01 | 21 |
| 7 | 0.22 | 0.04 | 29 | 0.16 | 0.04 | 21 |
| 14 | 0.39 | 0.09 | 29 | 0.20 | 0.04 | 21 |
| 21 | 0.32 | 0.05 | 29 | 0.18 | 0.05 | 21 |
| 28 | 0.24 | 0.07 | 29 | 0.14 | 0.05 | 21 |
| **56** | **0.38** | **0.09** | **29** | **0.14** | **0.08** | **21** |

**Mukhida 2007**

| Days after transplantation | Experiment | | | Control | | |
| --- | --- | --- | --- | --- | --- | --- |
|  | Mean | SD | N | Mean | SD | N |
| -10 (pre-injury) | 12.97 | 1.38 | 7 | 19.41 | 2.79 | 5 |
| 0 | 2.83 | 0.32 | 7 | 1.39 | 0.42 | 5 |
| 6 | 5.56 | 0.58 | 7 | 2.02 | 0.42 | 5 |
| 14 | 5.71 | 0.37 | 7 | 2.45 | 0.34 | 5 |
| **28** | **5.88** | **0.32** | **7** | **1.26** | **0.26** | **5** |
| 42 | 6.5 | 0.23 | 7 | 2.38 | 0.21 | 5 |

**2. Heat hyperalgesia threshold (s)**

**Dugan 2020**

| Days after transplantation | Experiment | | | Control | | |
| --- | --- | --- | --- | --- | --- | --- |
|  | Mean | SD | N | Mean | SD | N |
| -28 (pre-injury) | 17.41 | 0.65 | 12 | 16.27 | 0.98 | 12 |
| -21 | 12.54 | 1.40 | 12 | 13.36 | 2.00 | 12 |
| -14 | 9.85 | 1.30 | 12 | 14.27 | 1.24 | 12 |
| -7 | 6.35 | 1.45 | 12 | 9.91 | 1.24 | 12 |
| 0 | 5.56 | 0.81 | 12 | 6.09 | 0.49 | 12 |
| 7 | 7.70 | 1.14 | 12 | 5.01 | 0.65 | 12 |
| 14 | 8.29 | 1.02 | 12 | 5.39 | 1.24 | 12 |
| 21 | 9.20 | 0.81 | 12 | 5.23 | 1.45 | 12 |
| 28 | 9.16 | 1.63 | 12 | 4.91 | 1.02 | 12 |
| 35 | 9.59 | 1.24 | 12 | 4.32 | 1.34 | 12 |
| 42 | 9.81 | 1.45 | 12 | 4.64 | 1.14 | 12 |
| 49 | 10.08 | 1.34 | 12 | 3.89 | 0.92 | 12 |
| **56** | **10.6** | **1.25** | **12** | **3.25** | **1.18** | **12** |

**Eaton 2007**

| Days after transplantation | Experiment | | | Control | | |
| --- | --- | --- | --- | --- | --- | --- |
|  | Mean | SD | N | Mean | SD | N |
| -14 (pre-injury) | 14.37 | 0.39 | 5 | 13.56 | 0.36 | 5 |
| 0 | 10.76 | 0.41 | 5 | 9.95 | 0.36 | 5 |
| 7 | 13.60 | 0.88 | 5 | 9.63 | 0.26 | 5 |
| 14 | 13.61 | 0.24 | 5 | 10.34 | 0.34 | 5 |
| 21 | 13.76 | 0.32 | 5 | 9.95 | 0.27 | 5 |
| 28 | 13.94 | 0.22 | 5 | 10.36 | 0.26 | 5 |
| **35** | **13.90** | **0.37** | **5** | **9.11** | **0.59** | **5** |
| 42 | 13.8 | 0.14 | 5 | 10.6 | 0.45 | 5 |
| 49 | 13.67 | 0.26 | 5 | 8.97 | 0.56 | 5 |

**Fandel 2016**

| Days after transplantation | Experiment | | | Control | | |
| --- | --- | --- | --- | --- | --- | --- |
|  | Mean | SD | N | Mean | SD | N |
| -14 (Pre-injury) | - | - | - | 4.27 | 0.34 | 11 |
| 0 | - | - | - | 3.70 | 0.43 | 11 |
| 90 | 3.88 | 0.38 | 17 | 3.56 | 0.39 | 11 |
| **180** | **3.95** | **0.30** | **16** | **3.39** | **0.35** | **10** |

**Jergova 2012**

| Days after transplantation | Experiment | | | Control | | |
| --- | --- | --- | --- | --- | --- | --- |
|  | Mean | SD | N | Mean | SD | N |
| -7 (pre-injury) | 0.31 | 0.17 | 10 | 0.31 | 0.17 | 10 |
| 0 | -2.36 | 0.25 | 10 | -2.36 | 0.25 | 10 |
| **7** | **-0.43** | **0.34** | **10** | **-1.70** | **0.43** | **10** |
| 14 | -0.81 | 0.44 | 10 | -2.06 | 0.49 | 10 |
| 21 | -1.05 | 0.47 | 10 | -1.27 | 0.49 | 10 |
| 28 | -0.34 | 0.78 | 10 | -1.35 | 0.41 | 10 |
