# Supplementary material for: The Effect of GABAergic Cells Transplantation on Allodynia and Hyperalgesia in Neuropathic Animals: A Systematic Review With Meta-Analysis
Source: Front Neurol. 2022 Jul 4;13:900436. doi: 10.3389/fneur.2022.900436 (PMC9289294; doi:10.3389/fneur.2022.900436)
Supplement: eResult 2 — Support for judgement the SYRCLE's risk of bias tool. [file Data_Sheet_4.docx]

**eResult 2. support for judgement the SYRCLE's risk of bias tool**

Dugan 2020

Mutually beneficial effects of intensive exercise and GABAergic neural progenitor cell transplants in reducing neuropathic pain and spinal pathology in rats with spinal cord injury

| Item | Type of bias | Domain | Judgement | Support for judgement |
| --- | --- | --- | --- | --- |
| 1 | Selection bias | Sequence generation | Unclear | A key advantage of this compression SCI model is the ability to generate stable and long-term behaviorally measurable pain symptoms which allow for chronic testing (at least 12 weeks). In accordance with ARRIVE guidelines (Kilkenny et al., 2010), all animals were randomly assigned to the experimental groups. |
| 2 |  | Baseline characteristics | Unclear | All experiments were performed with adult, male Sprague Dawley rats obtained from Harlan Research Laboratories.  NPCs were resuspended in sterile saline vehicle for transplantation. NPCs or vehicle were injected into the lumbar spinal cord of male Sprague Dawley rats (250–300 g) at 4 weeks post-SCI. |
| 3 |  | Allocation concealment | Unclear | In accordance with ARRIVE guidelines (Kilkenny et al., 2010), all animals were randomly assigned to the experimental groups. |
| 4 | Performance bias | Random housing | Unclear | All animals were housed 2–3 per cage.  …free access to water and enrichment edibles. |
| 5 |  | Blinding | Unclear | Not mentioned. |
| 6 | Detection bias | Random outcome assessment | Unclear | The order of behavioral testing was randomized between experimental groups… |
| 7 |  | Blinding | Low | …and all behavioral evaluations were done in a blind mode. |
| 8 | Attrition bias | Incomplete outcome data | Unclear | Not mentioned. |
| 9 | Reporting bias | Selective outcome reporting | Low | - |
| 10 | Other bias | Other sources of bias | Unclear | Unable to determine whether there were new animals added to the control and experimental groups to replace drop-outs from the original population. |

Eaton 2007

Subarachnoid transplant of a human neuronal cell line attenuates chronic allodynia and hyperalgesia after excitotoxic spinal cord injury in the rat

| Item | Type of bias | Domain | Judgement | Support for judgement |
| --- | --- | --- | --- | --- |
| 1 | Selection bias | Sequence generation | Unclear | The animals then underwent an excitotoxic SCI (QUIS injection) to induce pain behaviors to tactile and thermal stimuli, as confirmed by a vigorous response to behavioral testing. Two weeks after injury, animals to be transplanted received a lumbar intrathecal cell graft with either viable hNT2.17 cells (differentiated for 2 weeks in vitro before transplant) or negative-control nonviable hNT2.17 cells. A third group of animals served as a control group and received only the QUIS injection but no transplant. A fourth group of animals received neither QUIS nor transplant and served as the naive controls. A fifth group of animals received intraspinal saline injections, rather than QUIS, and served as vehicle-only controls. |
| 2 |  | Baseline characteristics | Unclear | Not mentioned. |
| 3 |  | Allocation concealment | Unclear | Not mentioned. |
| 4 | Performance bias | Random housing | Low | Adult male Wistar-Furth rats (Harlan, Indianapolis, IN; approximately 200 to 250 g) were used for all behavioral experiments. The rats were housed 2 per cage with rat food and water ad libitum on a 12/12-hour light/dark cycle. |
| 5 |  | Blinding | Unclear | Not mentioned. |
| 6 | Detection bias | Random outcome assessment | Unclear | Not mentioned. |
| 7 |  | Blinding | Low | All behavioral testing was performed under blinded conditions to eliminate experimental bias, and data were analyzed and unblinded by the statistician at the end of the experiment. |
| 8 | Attrition bias | Incomplete outcome data | Unclear | Not mentioned. |
| 9 | Reporting bias | Selective outcome reporting | Unclear | Not mentioned. |
| 10 | Other bias | Other sources of bias | Unclear | Unable to determine whether there were new animals added to the control and experimental groups to replace drop-outs from the original population. |

Fandel 2016

Transplanted Human Stem Cell-Derived Interneuron Precursors Mitigate Mouse Bladder Dysfunction and Central Neuropathic Pain after Spinal Cord Injury

| Item | Type of bias | Domain | Judgement | Support for judgement |  |
| --- | --- | --- | --- | --- | --- |
| 1 | Selection bias | Sequence generation | Low | Then animals were randomized and baseline measurements, such as weight and pre-injury pain threshold, were determined. We used a website (https://www.random.org/sequences) to randomly allocate animals to treatment groups and to randomize the order of injury, the order of cell transplantation or vehicle injection, and the order of cystometry. |  |
| 2 |  | Baseline characteristics | Low |  |  |
| 3 |  | Allocation concealment | Low |  |  |
| 4 | Performance bias | Random housing | Low | Mice were housed in a specific pathogen-free facility at UCSF under a 12-hr light/12-hr dark cycle. Food and water were provided ad libitum. |  |
| 5 |  | Blinding | Low | The surgeon was blinded to baseline pain assessment outcomes. |  |
| 6 | Detection bias | Random outcome assessment | Unclear | All animals underwent randomization for injury allocation, and the spinal cord-injured animals were randomized again for treatment allocation. |  |
| 7 |  | Blinding | Low | All behavioral tests, including locomotor scoring and pain assessments, were conducted by investigators who were not involved in surgical procedures and were thus blinded to treatment (hESC-MGE transplantation or vehicle injection). |  |
| 8 | Attrition bias | Incomplete outcome data | Low | The following a priori criteria were used: ability of mice to extensively move one or both hind limb ankles (average BMS score > 1) on day 1 post-injury, and mice with bladder stones were excluded from bladder analysis (Figure S2). Any animals that died unexpectedly or were euthanized due to morbidity were excluded from any analyses. No additional animals were generated to replace animals that were removed based on the a priori criteria.  Animals with bladder stones were excluded from bladder analysis, and an equal number of spinal cord-injured animals in each treatment arm developed bladder stones. |  |
| 9 | Reporting bias | Selective outcome reporting | Low | - |  |
| 10 | Other bias | Other sources of bias | Low | - |  |

Hwang 2016

Intrathecal Transplantation of Embryonic Stem Cell-Derived Spinal GABAergic Neural Precursor Cells Attenuates Neuropathic Pain in a Spinal Cord Injury Rat Model

| Item | Type of bias | Domain | Judgement | Support for judgement |
| --- | --- | --- | --- | --- |
| 1 | Selection bias | Sequence generation | Low | Fourteen rats included were randomly allocated into saline or cell injection group using random allocation software. |
| 2 |  | Baseline characteristics | Unclear | Not mentioned. |
| 3 |  | Allocation concealment | Unclear | Not mentioned. |
| 4 | Performance bias | Random housing | Low | Animals were housed under a 12 h/12 h light/dark cycle (08:00–20:00) with free access to water and food. |
| 5 |  | Blinding | Unclear | Not mentioned. |
| 6 | Detection bias | Random outcome assessment | Unclear | Not mentioned. |
| 7 |  | Blinding | Low | All behavioral tests in this study were assessed by an experimenter who was blinded to the treatment groups. |
| 8 | Attrition bias | Incomplete outcome data | Low | - |
| 9 | Reporting bias | Selective outcome reporting | Low | - |
| 10 | Other bias | Other sources of bias | Low | - |

Jergova 2012

Analgesic Effect of Recombinant GABAergic Cells in a Model of Peripheral Neuropathic Pain

| Item | Type of bias | Domain | Judgement | Support for judgement |
| --- | --- | --- | --- | --- |
| 1 | Selection bias | Sequence generation | Unclear | Not mentioned. |
| 2 |  | Baseline characteristics | Unclear | Not mentioned. |
| 3 |  | Allocation concealment | Unclear | Not mentioned. |
| 4 | Performance bias | Random housing | Unclear | Animals were housed two per cage with free access to food and water and a 12-h light/dark cycle. Experimental procedures were reviewed and approved by the University of Miami Institutional Animal Care and Use Committee (IACUC) and followed the recommendations of the Guide for the Care and Use of Laboratory Animals (National Research Council).  One week after CCI animals showing changes in the reaction to thermal or mechanical stimuli, (behavioral testing details below) were used for intraspinal injection of cells (n=38) or equal volume of saline vehicle (n=34). |
| 5 |  | Blinding | Unclear | Not mentioned. |
| 6 | Detection bias | Random outcome assessment | Unclear | Not mentioned. |
| 7 |  | Blinding | Low | All behavioral tests were performed by a trained person blinded to the experimental treatment. The same person always performed a given test to reduce variability. |
| 8 | Attrition bias | Incomplete outcome data | Unclear | - |
| 9 | Reporting bias | Selective outcome reporting | Low | - |
| 10 | Other bias | Other sources of bias | Low | - |

Jergova 2016a

Recombinant neural progenitor transplants in the spinal dorsal horn alleviate chronic central neuropathic pain

| Item | Type of bias | Domain | Judgement | Support for judgement |
| --- | --- | --- | --- | --- |
| 1 | Selection bias | Sequence generation | Unclear | Not mentioned. |
| 2 |  | Baseline characteristics | Unclear | Not mentioned. |
| 3 |  | Allocation concealment | Unclear | Not mentioned. |
| 4 | Performance bias | Random housing | Low | Male Sprague-Dawley rats were used for the spinal cord injury, implantation of intrathecal catheter and intraspinal injections (140–160g at the time of the first surgery); Animals were housed two per cage with free access to food and water in 12 h light/dark cycle. |
| 5 |  | Blinding | Unclear | Not mentioned. |
| 6 | Detection bias | Random outcome assessment | Unclear | Not mentioned. |
| 7 |  | Blinding | Low | Behavioral responses were recorded by observers blinded to experimental treatments. |
| 8 | Attrition bias | Incomplete outcome data | Unclear | Insufficient data to judge  …total n=62; n’s for individual experiments and treatment groups appear in figure legends.  n=6/saline; n=10/transplant*3, |
| 9 | Reporting bias | Selective outcome reporting | Low | - |
| 10 | Other bias | Other sources of bias | Low | - |

Kim 2010

Transplantation of GABAergic neurons from ESCs attenuates tactile hypersensitivity following spinal cord injury

| Item | Type of bias | Domain | Judgement | Support for judgement |
| --- | --- | --- | --- | --- |
| 1 | Selection bias | Sequence generation | Unclear | Not mentioned. |
| 2 |  | Baseline characteristics | Unclear | Not mentioned. |
| 3 |  | Allocation concealment | Unclear | Not mentioned. |
| 4 | Performance bias | Random housing | Low | The rats were housed in groups of four, provided with food and water ad libitum under a 12-hour light/dark cycle, and allowed to acclimate for a week before surgery and behavioral testing. All animal experiments were carried out in accordance with NIH regulations for animal care and approved by the Institutional Animal Care and Use Committee of Yonsei University College of Medicine. |
| 5 |  | Blinding | Unclear | Not mentioned. |
| 6 | Detection bias | Random outcome assessment | Unclear | Not mentioned. |
| 7 |  | Blinding | Low | Behavioral testing, neuronal activity recording, and cell counting were performed by investigators blinded to the animal treatments. |
| 8 | Attrition bias | Incomplete outcome data | Unclear | However, autophagic behavior (mild self- inflicted injury on hindlimb) was observed in some rats that received cell transplantation (5 of 45 rats) with a similar incidence in vehicle-injected control rats (10 of 87 rats). As we had not been able to examine the pain-like response on injured feet of rats with autophagic behavior, those rats were excluded from further analysis in the present study. |
| 9 | Reporting bias | Selective outcome reporting | Low | - |
| 10 | Other bias | Other sources of bias | Low | - |

Li 2018

人胚胎干细胞来源的脊髓GABA能神经前体细胞移植治疗脊髓损伤大鼠神经病理性疼痛和痉挛的疗效研究

| Item | Type of bias | Domain | Judgement | Support for judgement |
| --- | --- | --- | --- | --- |
| 1 | Selection bias | Sequence generation | Unclear | 大鼠随机分成假手术组、脊髓损伤组、细胞移植组。 |
| 2 |  | Baseline characteristics | Unclear | Not mentioned. |
| 3 |  | Allocation concealment | Unclear | Not mentioned. |
| 4 | Performance bias | Random housing | Low | 大鼠体重为 200-220 g。大鼠成对饲养于动物实验中心温度控制动物饲养体系。提供充足的水和食物供动物自由摄取，保持 12 h/12 h 夜节律。所有动物操作均在华中科技大学同济医学院附属同济医院动物管理中心批准和指导下进行，符合动物伦理要求。 |
| 5 |  | Blinding | Unclear | Not mentioned. |
| 6 | Detection bias | Random outcome assessment | Unclear | Not mentioned. |
| 7 |  | Blinding | Low | 行为学评估人员不了解大鼠的分组处理。 |
| 8 | Attrition bias | Incomplete outcome data | Unclear | Not mentioned. |
| 9 | Reporting bias | Selective outcome reporting | Unclear | Not mentioned. |
| 10 | Other bias | Other sources of bias | Unclear | Unable to determine whether there were new animals added to the control and experimental groups to replace drop-outs from the original population and whether the study was free of the inappropriate influence of funding angencies. |

Manion 2020

Human induced pluripotent stem cell-derived GABAergic interneuron transplants attenuate neuropathic pain

| Item | Type of bias | Domain | Judgement | Support for judgement |
| --- | --- | --- | --- | --- |
| 1 | Selection bias | Sequence generation | Unclear | assignment to treatment groups was performed pseudorandomly by an experimenter blind to behaviour data and health status. |
| 2 |  | Baseline characteristics | Unclear | Not mentioned. |
| 3 |  | Allocation concealment | Low | The group assignments were performed before injections by an investigator blind to the behavioural results and health status of all the animals in a pseudorandom manner. |
| 4 | Performance bias | Random housing | Low | Mice were housed on a 12hr light dark cycle and provided with standard chow and water ad libitum at all stages. All mice were maintained in a specific pathogen free facility and aseptic technique was used for all handling and experimentation. |
| 5 |  | Blinding | Low | All animal experiments were performed blind to treatment |
| 6 | Detection bias | Random outcome assessment | Unclear | Not mentioned. |
| 7 |  | Blinding | low | All behaviour was performed by a single male investigator. All experiments involving animals were performed blind (the surgeon performing the laminectomy, SNI, and behaviour was unaware of group assignments). |
| 8 | Attrition bias | Incomplete outcome data | Low | A total of 50 mice  Normalised von Frey thresholds of injured mice after nerve injury and spinal transplantation of GABAergic neurons (n = 21 vehicle, 29 GABAergic neurons). |
| 9 | Reporting bias | Selective outcome reporting | Low | We did not exclude any outliers; however, any mouse that trapped its paw was not measured for von Frey at that attempt, we performed all calculations including and excluding these mice, and this did not affect the overall result. |
| 10 | Other bias | Other sources of bias | Low | - |

Mukhida 2007

Spinal GABAergic Transplants Attenuate Mechanical Allodynia in a Rat Model of Neuropathic Pain

| Item | Type of bias | Domain | Judgement | Support for judgement |
| --- | --- | --- | --- | --- |
| 1 | Selection bias | Sequence generation | Unclear | Postligation, rats were randomly assigned to treatment groups and received intraspinal transplants of fetal striatal primordia cells derived from transgenic green fluorescent protein (GFP) mice (n = 7), HNPCs differentiated into a GABAergic phenotype (n = 7), undifferentiated HNPCs (n = 7), or cell suspension medium only (n = 5). |
| 2 |  | Baseline characteristics | Unclear | Not mentioned. |
| 3 |  | Allocation concealment | Unclear | Not mentioned. |
| 4 | Performance bias | Random housing | Low | Twenty-six female Wistar rats (Charles River Laboratories, Saint Constant, Quebec, Canada, http://www.criver.com) weighing 175–200 g were used and housed in pairs in a temperature/humidity-controlled room on a 12-hour light/dark cycle with access to food and water ad libitum when behavioral tests were not being performed. The experiments were conducted in accordance with the guidelines of the Canadian Council on Animal Care. |
| 5 |  | Blinding | Unclear | Ten days postligation, animals received transplants of either 200,000 fetal striatal primordia GFP cells, HNPCs differentiated into a GABAergic phenotype, undifferentiated HNPCs, or cell suspension medium only into the spinal cord. |
| 6 | Detection bias | Random outcome assessment | Unclear | Not mentioned. |
| 7 |  | Blinding | Low | The same investigator performed the test for all animals at all time points and was observed by another investigator to ensure consistency. Both investigators were blinded to the treatment. |
| 8 | Attrition bias | Incomplete outcome data | Low | - |
| 9 | Reporting bias | Selective outcome reporting | Low | - |
| 10 | Other bias | Other sources of bias | Low | - |
